# Topical Therapy with Antisense Tumor Necrosis Factor Alpha Using Novel β-Glucan-Based Drug Delivery System Ameliorates Intestinal Inflammation

**DOI:** 10.3390/ijms21020683

**Published:** 2020-01-20

**Authors:** Hideto Sakisaka, Hidetoshi Takedatsu, Keiichi Mitsuyama, Shinichi Mochizuki, Kazuo Sakurai, Shotaro Sakisaka, Fumihito Hirai

**Affiliations:** 1 Department of Gastroenterology and Medicine, Fukuoka University Faculty of Medicine, 7-45-1 Nanakuma, Jonan-ku, Fukuoka 814-0180, Japan; mm050040@yahoo.co.jp (H.S.); sakisaka@fukuoka-u.ac.jp (S.S.); fuhirai@cis.fukuoka-u.ac.jp (F.H.); 2 Division of Gastroenterology, Department of Medicine, Kurume University School of Medicine, Kurume 830-0011, Japan; ibd@med.kurume-u.ac.jp; 3 Department of Life and Environment Engineering, The University of Kitakyushu, Kitakyushu 808-0135, Japan; mochizuki@kitakyu-u.ac.jp (S.M.); sakurai@kitakyu-u.ac.jp (K.S.)

**Keywords:** inflammatory bowel disease, antisense therapy, drug delivery system

## Abstract

Anti-tumor necrosis factor alpha (TNF-α) antibodies are effective in patients with inflammatory bowel disease (IBD). However, the effect is not optimal because a sufficient concentration of antibodies cannot be maintained at the site of inflammation. Thus, a macromolecular complex was developed with schizophyllan (SPG) and antisense oligonucleotides. In the present study, an SPG-antisense TNF-α complex was prepared, and its therapeutic efficacy was examined using a dextran sodium sulfate (DSS)-induced colitis model. The TNF-α production in CD11b+ macrophages significantly increased in the colon of DSS-treated mice. Dectin-1, a receptor of SPG, binds with SPG and is subsequently taken into the cells via phagocytosis. The expression of dectin-1 by CD11b+ macrophages significantly increased in DSS-treated mice. Flow cytometry revealed that the uptake of SPG-antisense TNF-α in the macrophages was efficient. TNF-α production was suppressed significantly by SPG-antisense TNF-α in vitro, which was administered via enema to evaluate its efficacy. The intrarectal administration of SPG-antisense TNF-α ameliorated the intestinal inflammation. In this study, we showed that the delivery system that conjugates SPG and antisense can have higher therapeutic efficacy. Thus, the new therapeutic approach presented in this study may be used in the management of IBD.

## 1. Introduction

Inflammatory bowel disease (IBD) is a chronic inflammatory condition of the gastrointestinal tract. The two major IBD phenotypes are ulcerative colitis (UC) and Crohn’s disease (CD) [1]. The pathogenesis of IBD is complex; however, the dysregulation of the intestinal immune system plays a key role in the development of the condition [2]. In a previous study, an increased level of TNF-α was detected in the mucosa of patients with IBD, and the intestinal inflammatory process shifted to Th1 during cytokine production [3,4]. In animal models, anti-tumor necrosis factor alpha (anti-TNF-α) antibodies (Abs) improved intestinal inflammation, indicating that they effectively increased cytokine production by Th1 cells in chronic inflammatory disease [5]. Thus, TNF-α has a pathogenic role as anti-TNF-α Abs significantly improved intestinal inflammation in IBD [6]. Ant-TNF-α Abs were then widely used in patients with IBD.

Although anti-TNF-α Abs were found to be effective in the treatment of IBD [7], high doses are required to improve intestinal inflammation. Therefore, the incidence of adverse events, such as tuberculosis, lymphoma, and the development of antibodies against anti-TNF-α Abs, is increasing [8]. Thus, the strategies used in biological treatment must be improved to downregulate TNF-α in IBD. Recently, several studies have shown that small interfering RNA (siRNA) and antisense oligonucleotides (ASOs) that inhibit the production of TNF-α are useful for the treatment of IBD [9,10,11]. However, previous studies investigated the effect of siRNA and ASOs administered orally and subcutaneously, and only few reports provided local treatment via enema.

**Figure 1:**
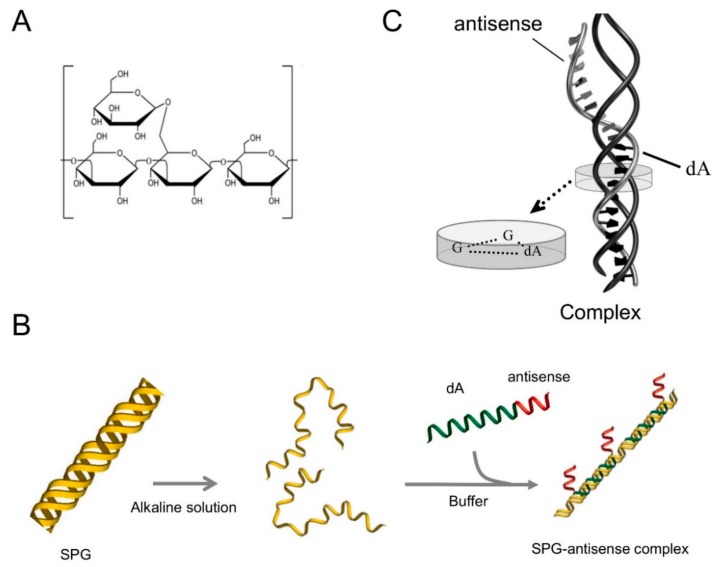
Schematic illustration showing a complex structure composed of antisense oligonucleotides (ASOs) with a dA tail and schizophyllan (SPG). (**A**) Chemical structure of SPG. (**B**) Schematic illustration of the SPG–ASO complex. (**C**) A triple-stranded complex was formed from one DNA and two SPG strands.

Schizophyllan (SPG), which belongs to the β-(1-3) glucan family (Figure 1A), forms a triple helix in a neutral solution. This triple helix changes to three single chains in an alkaline solution (Figure 1B). The single chain of SPG present in alkaline solutions reverts to a triple helix via hydrophobic and hydrogen bonding interactions in neutral solutions. During this process, we found that polynucleotides built on poly(dA) form a stoichiometric complex with two single chains of SPG (Figure 1B,C) [12]. By contrast, a short antisense oligonucleotide does not form a complex with SPG. In this study, an SPG-based drug delivery system (DDS) with oligonucleotides comprising ASOs and poly(dA) was developed to deliver ASOs to the target sites. SPG has several advantages. The SPG used in this study was medical grade [13] and was up taken in macrophages and dendritic cells (DCs) via phagocytosis [14]. Furthermore, previous studies showed that Dectin-1, which was known to be the receptor of SPG, had a key role in the phagocytosis of SPG in macrophages [15,16].

In our study, the topical administration of SPG-antisense TNF-α suppressed TNF-α production by macrophages and significantly improved dextran sodium sulfate (DSS)-induced colitis.

## 2. Results

### 2.1. Cytokine Production in the Mucosa of DSS-Treated Mice

**Figure 2:**
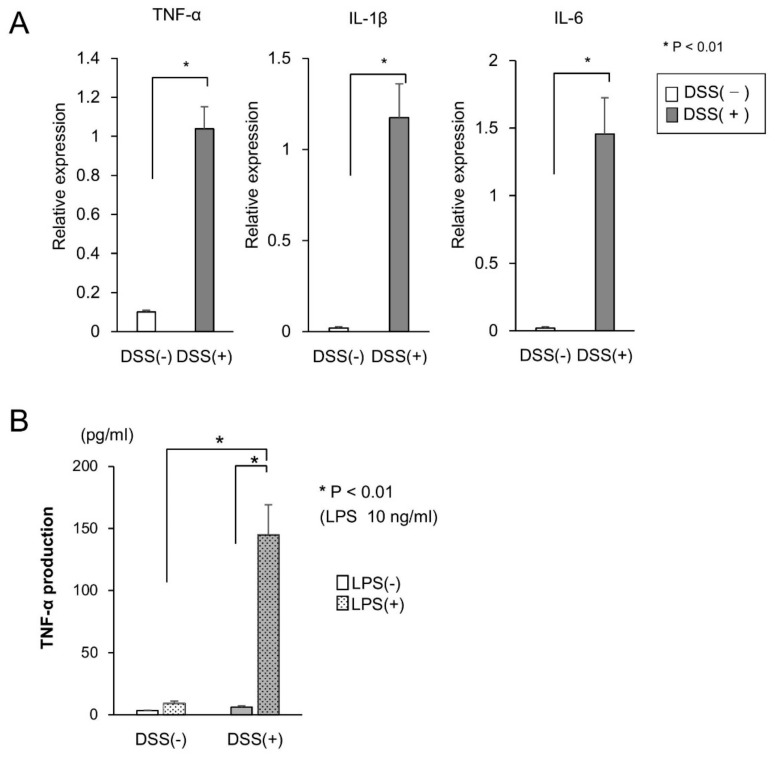
Tumor necrosis factor alpha (TNF-α) is up-regulated in dextran sodium sulfate (DSS)-induced colitis. (**A**) Interleukin (IL)-1, IL-6, and tumor necrosis factor alpha (TNF-α) mRNA expressions in colon specimens were assessed using real-time polymerase chain reaction (*n* = 5 per group). Data were normalized to the expression of glyceraldehyde-3-phosphate dehydrogenase (GAPDH) mRNA. (**B**) CD11b+ cells, isolated from the lamina propria in the dextran sodium sulfate-treated mice and untreated mice, were cultured with 10 ng/mL lipopolysaccharide (LPS). The production of TNF-α was measured using an enzyme-linked immunosorbent assay. Data were presented as the mean of the three independent experiments.

The expressions of TNF-α and other proinflammatory cytokines, such as interleukin (IL)-1β and IL-6, in the mucosa of DSS-treated mice were examined via real-time polymerase chain reaction (PCR). The expression of these proinflammatory cytokines in the DSS-treated mice increased significantly (Figure 2A). Since the expression of TNF-α in the mucosa of DSS-treated mice increased, we examined the production of TNF-α in CD11b+ cells isolated from lamina propria (LP) cells. The production of TNF-α in CD11b+ cells cultured with 10 ng/mL LPS was significantly higher in DSS-treated mice than in DSS-untreated mice (Figure 2B). Since CD11b+ cells produced high amounts of TNF-α in the mucosa of DSS-treated mice, we targeted TNF-α in CD11b+ cells to improve colitis.

### 2.2. The Expression of Dectin-1 in CD11b+ Cells Significantly Increased in the Mucosa of DSS-Treated Mice

**Figure 3:**
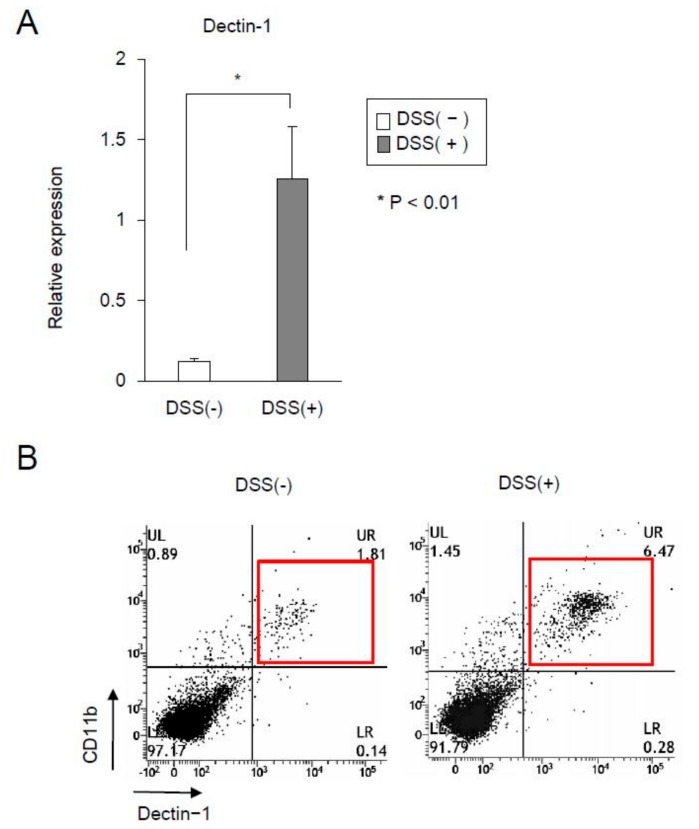
The expression of dectin-1 in the receptor of schizophyllan increased in dextran sodium sulfate-induced acute colitis. (**A**) Dectin-1 mRNA expression in the colon was evaluated using real-time polymerase chain reaction. Data were normalized to the expression of glyceraldehyde-3-phosphate dehydrogenase (GAPDH) mRNA. (**B**) Dectin-1 and CD11b expressions in the lamina propria were analyzed via fluorescence activated cell sorter (FACS) analysis. Data were presented as the mean of the three independent experiments.

Dectin-1 is a pathogen pattern-recognition receptor (PRR) in macrophages and DCs, and it binds with β-glucans, including SPG [17]. The expressions of dectin-1 in DSS-untreated and DSS-treated mice were examined. The results showed that the expression in the mucosa was significantly higher in DSS-treated mice than in DSS-untreated mice (Figure 3A). Subsequently, we examined the expression of dectin-1 in CD11b+ cells of LP. Fluorescence activated cell sorter (FACS) analysis showed that the expression of dectin-1 in CD11b+ cells increased in the LP of DSS-treated mice compared with DSS-untreated mice (Figure 3B). In this study, since most CD11b+ cells expressed dectin-1, the SPG-based delivery system was assumed to be taken up into CD11b+ cells via dectin-1.

### 2.3. SPG-Antisense TNF-α Inhibited the Production of TNF-α in CD11b+ Cells

**Figure 4:**
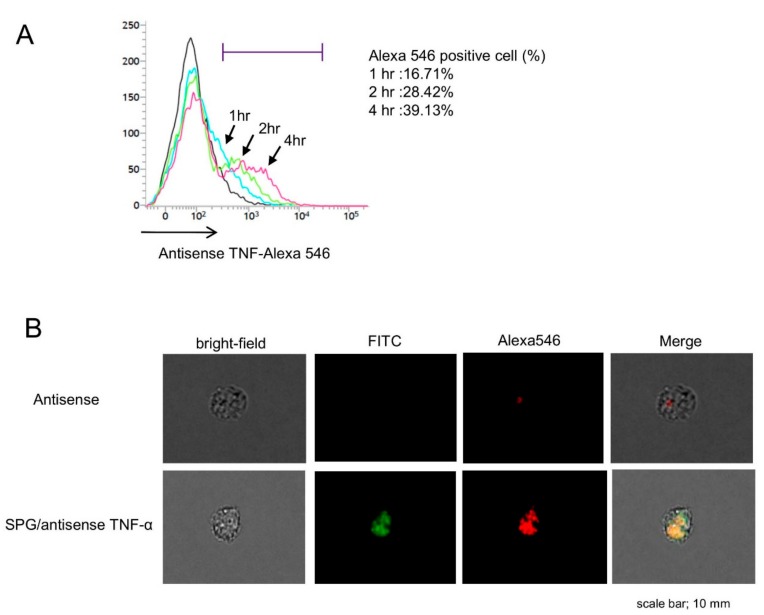
Schizophyllan (SPG)-antisense tumor necrosis factor alpha (TNF-α) inhibited the production of TNF-α induced by lipopolysaccharide (LPS) in vitro. (**A**) fluorescence activated cell sorter (FACS) analysis revealed that SPG-antisense TNF-α labeling with Alexa Fluor 546 (Alexa 546) was taken up into CD11b+ cells in a time-dependent manner. (**B**) Immunofluorescence in CD11b+ cells was performed by labeling antisense TNF-α with Alexa546 and the SPG with fluorescein isothiocyanate (FITC) CD11b+ cells more effectively took up the SPG-antisense TNF-α compared with antisense TNF-α alone.

**Figure 5:**
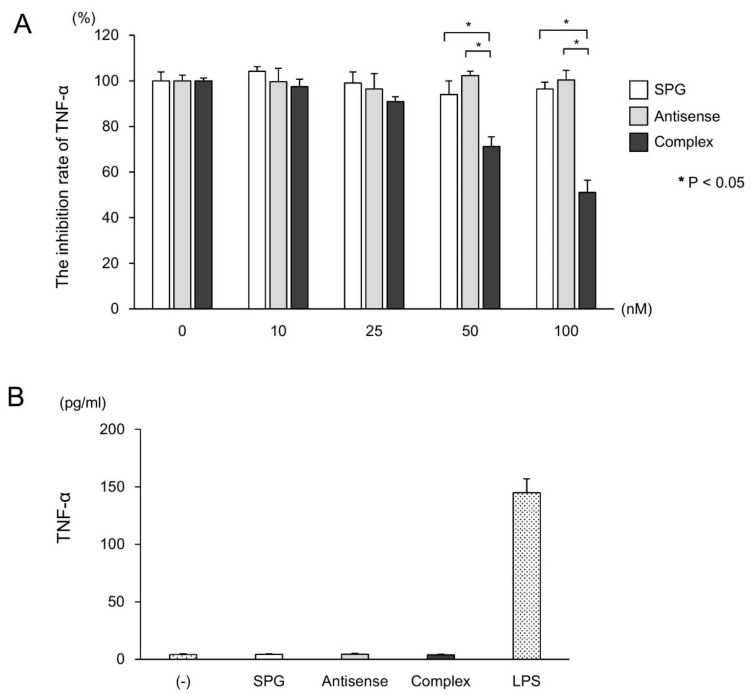
Schizophyllan (SPG)-antisense tumor necrosis factor alpha (TNF-α) inhibits the production of TNF-α in CD11b cells. (**A**) CD11b+ cells in the lamina propria were cultured with several concentrations of SPG-antisense TNF-α (complex), antisense TNF-α, and SPG as a control. After 10 h, 10 ng/mL lipopolysaccharide (LPS) was added under each condition, and the cells were cultured for 24 h. The production of TNF-α was measured using an enzyme-linked immunosorbent assay (*n* = 5 per group). Data were presented as means ± standard deviation. (**B**) The CD11b+ cells were cultured with SPG, antisense TNF-α, SPG-antisense TNF-α, and LPS for 24 h. The production of TNF-α was measured using an enzyme-linked immunosorbent assay (*n* = 5 per group).

We examined the uptake rate of SPG-antisense TNF-α at different time points (Figure 4A). The CD11b+ cells in the LP were cultured with SPG-antisense TNF-α at 0, 1, 2, and 4 h. FACS analysis revealed that approximately 40% was taken up into the macrophages for 4 h after the administration of the complex. Furthermore, we performed immunofluorescence to examine whether the SPG-antisense TNF-α was taken up into CD11b+ cells in vitro (Figure 4B). Antisense TNF-α and SPG were labeled with Alexa Fluor 546 (Alexa546) and fluorescein isothiocyanate (FITC), respectively. As shown in Figure 4B, a large number of Alexa546 and FITC double positive CD11b+ cells was detected in the SPG-antisense TNF-α group. This results showed that the SPG-antisense TNF-α was taken up by the CD11b+ cells in large numbers compared with antisense TNF-α without DDS. Furthermore, we investigated whether SPG-antisense TNF-α inhibited the production of TNF-α in CD11b+ cells. The CD11b+ cells with increasing concentrations of SPG-antisense TNF-α were cultured with 10 ng/mL LPS in vitro, and their TNF-α production was measured. SPG-antisense TNF-α significantly inhibited the production of TNF-α depending on the concentration of SPG-antisense TNF-α (Figure 5A). Antisense TNF-α and SPG did not inhibit the production of TNF-α (Figure 5B). Furthermore, we investigated whether SPG and SPG-antisense TNF-α stimulated CD11b+ cells via dectin-1 to induce TNF-α production. The results showed that SPG and SPG-antisense TNF-α did not produce TNF-α in CD11b+ cells.

### 2.4. Administration of SPG-Antisense TNF-α Improved Colitis in DSS-Treated Mice

**Figure 6:**
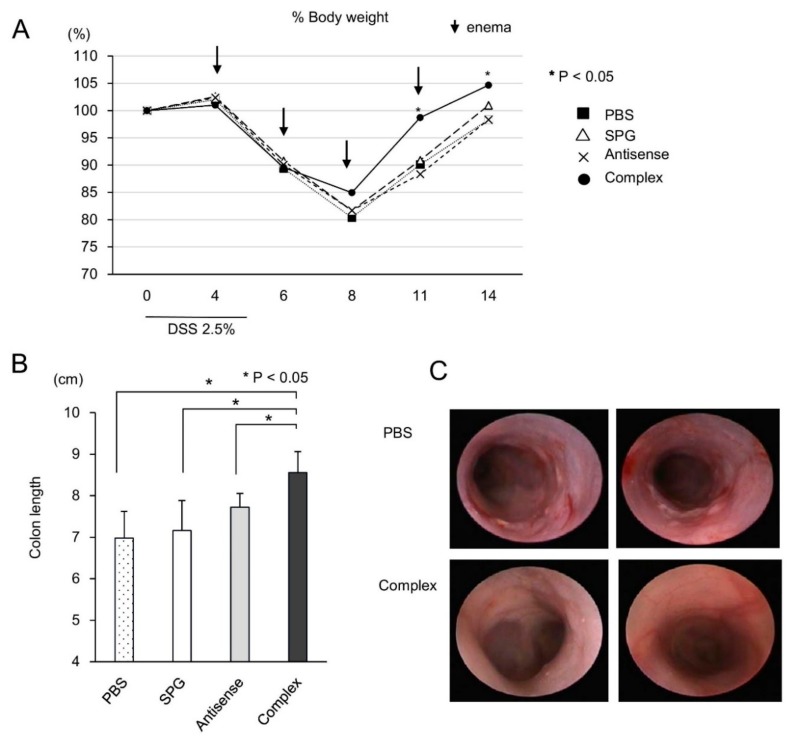
Attenuation of dextran sodium sulfate (DSS)-induced colitis via the administration of schizophyllan (SPG)-antisense tumor necrosis factor alpha (TNF-α). A total of 0.2 mg/kg SPG-antisense TNF-α (complex), antisense TNF-α (antisense), SPG, and a phosphate buffer solution used as a control, were administered via enema twice weekly in mice receiving DSS (*n* = 8 per group). (**A**) Body weights are presented as percentage of the initial weight on day 0. (**B**) The length of the colon from the terminal ileum to the rectum. (**C**) Endoscopic findings of the colon.

**Figure 7:**
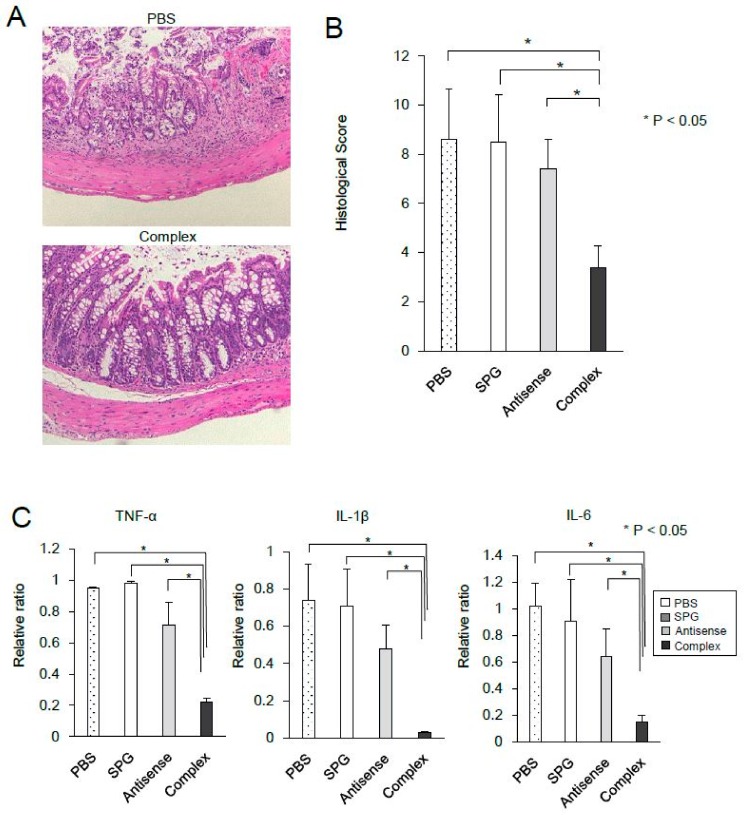
Administration of schizophyllan (SPG)-antisense tumor necrosis factor alpha (TNF-α) inhibited cytokines in dextran sodium sulfate (DSS)-induced colitis. (**A**) Hematoxylin and eosin staining of the colon (original magnification: ×100). (**B**) Evaluation of the histological scores. (**C**) TNF-α, Interleukin (IL)-1, and IL-6 mRNA expressions in the colon were assessed using real-time polymerase chain reaction (*n* = 5 per group). Data were normalized to the expression of glyceraldehyde-3-phosphate dehydrogenase (GAPDH) mRNA.

The results showed that SPG-antisense TNF-α inhibited the production of TNF-α in vitro. To investigate the therapeutic effect of SPG-antisense TNF-α in vivo, 0.2 mg/kg SPG-antisense TNF-α was administered via enema twice per week in DSS-treated mice (Figure 6A); 0.2 mg/kg SPG-antisense TNF-α is equal to 50 nM. The therapeutic effect of SPG-antisense TNF-α was evaluated on day 14 to evaluate the severity of the disease and cytokine production. The topical administration of SPG-antisense TNF-α significantly inhibited weight loss and colon shortening (Figure 6A,B). Upon endoscopy, edema and erosion in the intestine improved after treatment with SPG-antisense TNF-α (Figure 6C). A histological examination also revealed a significant improvement in mucosal damage and inflammation after treatment with SPG-antisense TNF-α (Figure 7A,B). The production of TNF-α and other proinflammatory cytokines in the colon was inhibited by SPG-antisense effectively inhibited the production of TNF-α in CD11b+ cells, and improved intestinal inflammation.

## 3. Discussion

TNF-α is produced by macrophages and T cells, and is a key cytokine in the development of inflammation. Moreover, it plays a role in the pathogenesis of IBD since the level of TNF-α increases in the serum of patients with UC and CD [18]. Furthermore, patients with IBD have increased levels of TNF-α in the inflamed mucosa [3,19] and TNF-α produced by CD14+ macrophages [20]. Although the rate of clinical response to anti-TNF-α Abs is approximately 60%, lack of response and disease relapse are commonly observed during maintenance therapy [21,22,23]. Moreover, in the study of Olesen et al. 50% of the patients did not, for several reasons, respond during therapy [24]. This is mainly attributed to the development of neutralizing Abs against therapeutic protein [25,26]. The complex produced in this study is nano-sized and has a minimal effect when it acts locally. Therefore, it does not require large amounts of protein, similar to antibody therapy. However, the production of an antibody is considered challenging.

Macrophages and DCs recognize pathogens such as fungi and mycobacteria using PRRs, activate innate immunity, and eliminate the pathogens. Dectin-1 is a type II C-type lectin receptor that belongs to the PRRs. It is highly expressed in macrophages and DCs, and binds with the carbohydrate structures of pathogens [27,28]. Previous studies have shown that dectin-1 is the receptor for β-1, 3-linked glucans and that it is a protective host against fungi [29,30]. The level of dectin-1 increased in the inflamed mucosa, but not in a disease-specific manner [31]. In fact, the expression of dectin-1 in intestinal macrophages increased in the inflamed mucosa in DSS colitis. Therefore, ASO with SPG is efficiently taken up by dectin-1-positive macrophages and DCs at the site of inflammation. The dectin-1 signaling pathway directly activated nuclear factor kappa and induced proinflammatory cytokines, such as TNF-α and IL-6 [32]. However, the mechanism underlying the effect of dectin-1 in IBD remains unclear. Heinsbroek et al. have shown that the lack of dectin-1 did not improve intestinal inflammation in the colitis mouse model, although some differences were observed in the innate immune response to gut flora between dectin-1-deficient mice and normal mice [33]. In our study, SPG alone and SPG-antisense TNF-α did not induce the production of TNF-α in CD11b+ macrophages. In addition, they did not exacerbate DSS-induced colitis. Our study showed that the SPG-based DDS did not induce the production of proinflammatory cytokines and did not cause intestinal inflammation via Dectin-1, both in vivo and in vitro.

ASOs have been used in patients with IBD to inhibit specific targets, including Smad7, intercellular adhesion molecule (ICAM)-1, and toll-like receptor (TLR) 9. Mongersen, a Smad7 ASO targeting RNA, encoded in the 107–128 DNA region, was developed for CD therapy. In a phase II clinical trial of patients with CD, Mongersen significantly improved Crohn’s Disease Activity Index scores [34]. Although therapeutic effects were expected, a phase III clinical trial did not show such treatment to be notably effective. The expression of ICAM-1, which is an adhesion molecule mediating the adhesion and migration of leucocytes from the blood to the intestine, increased in the endothelial cells in IBD [35,36]. Alicaforsen is a 20-base phosphorothioate ASO that hybridizes the mRNA of ICAM-1 [37]. Although Alicaforsen did not have a therapeutic effect in patients with CD, it was found to be effective in patients with UC when administered via enema, according to several studies [38]. The expression of TLR9, which increases in the inflamed intestine, has been considered an important pathogen in UC [39]. Cobitolimod is an ASO that is recognized by TLR9. Currently, in a phase II clinical study of cobitolimod in patients with moderate to severe UC, enema was performed. Treatment with ASO has also been used clinically; however, its efficacy was not confirmed in multicenter clinical trials.

Based on these results, the use of ASO and siRNA has several disadvantages. Hydrolysis mediated by deoxyribonuclease causes the instability of ASO and siRNA. Thus, additional strategies are required for them to reach the intestine and stay in the intestinal mucosa, despite inflammation causing a loss of mucous gel layers and the disruption of the epithelial cell barrier in the intestine. The administration of ASOs with SPG directly to an inflammatory site forming an ulcer effectively acts on the inflammatory site and does not affect the whole body. The SPG-based antisense delivery system had several advantages. The SPG complex was stable in vivo and did not dissolve in the presence of deoxyribonuclease, and it was effectively taken up by macrophages via dectin-1 and phagocytosis. Therefore, the topical administration of the SPG-antisense TNF-α significantly inhibited the production of TNF-α by the macrophages and improved intestinal inflammation. These complexes are considered effective against human IBD when administered orally in the form of PH-dependent capsules or via enema. Thus, the efficacy and safety of SPG-based antisense therapy in individuals with IBD must be further investigated.

## 4. Materials & Methods

### 4.1. Preparation of SPG-Antisense TNF-α

SPG (*M*_w_ = 1.5 × 10^5^, via gel-permeation chromatography) was provided by Mitsui Sugar Co., Ltd. (Tokyo, Japan). ASO for TNF-α (AACCCATCGGCTGGCACCAC-(dA)_60_) was synthesized at FASMAC Co., Ltd. (Kanagawa, Japan) and purified using high-performance liquid chromatography. SPG can dissociate from a triple helix to a single chain in 0.25 N NaOH for 3–5 days. The single chains of SPG were mixed with antisense TNF-α in a phosphate buffer solution (pH 7.4) and were stored overnight at 4 °C. The final DNA concentrations of the complex were measured using ultraviolet absorbance. The formation of SPG-based antisense complex was confirmed via polyacrylamide gel electrophoresis.

### 4.2. Mice

C57BL/6 mice were purchased from SLC (Fukuoka, Japan) and the Kyudo Laboratory (Saga, Japan). All experiments were performed under specific pathogen-free conditions at Kurume University and Fukuoka University. The mice were used according to the approved protocols of the Animal Care and Use Committees of Kurume University Animal (number 028/2015, approval data 16 March 2015) and Fukuoka University (number 1850/2017, approval date 18 January 2017).

### 4.3. DSS-Induced Colitis Model

Colitis was induced with 3% (*w*/*v*) DSS (40,000–50,000 MW) (MP Biomedicals, Irvine, CA, USA) drinking water. Eight-week-old female mice were provided with 3% DSS water for 5 days. The body weights of the mice were assessed to evaluate the development of colitis. They were sacrificed on day 14 and the colon was removed and used in the experiments. A part of the colon was fixed in 4% formaldehyde and was stained with hematoxylin and eosin. The histology was scored, as previously described [40]. Inflammation (I) was scored as 0 = none, 1 = mild, 2 = moderate, and 3 = severe; extent (E) as 0 = none, 1 = mucosa, 2 = mucosa and submucosa, and 3 = transmural; regeneration (R) as 0 = complete regeneration, 1 = almost complete regeneration, 2 = regeneration with crypt depletion, 3 = surface epithelium not intact, and 4 = no tissue repair; crypt damage (C) as 0 = none, 1 = 1/3 of basal damage, 2 = 2/3 of basal damage, 3 = only the surface epithelium is intact, and 4 = loss of the entire crypt and epithelium; and percent involvement (P) as 1 = 1–25%, 2 = 26–50%, 3 = 51–75%, and 4 = 76–100%. The severity of colitis was evaluated using the total histological scores I+E+R+C+P.

### 4.4. Cell Culture and Cytokine Assay

For the cell culture, mononuclear cells were isolated from the LP in the colon. The intestinal epithelial cells were removed from the colon with 1 mmol/L EDTA. Then, colonic tissues were shredded and digested by 100 U/mL of collagenase type 2. The LP cells were passed through a 100 µm nylon membrane and were purified using a 45%/72% Percoll gradient (GE Healthcare, Piscataway, NJ, USA). The CD11b+ cells were isolated via magnetic activated cell sorting using anti-CD11b MicroBeads (Miltenyi Biotec, Auburn, CA, USA). Moreover, 1 × 10^6^ CD11b+ cells were cultured in 96-well plates with 10 ng/mL LPS (Sigma-Aldrich, St. Louis, MO, USA). The production of TNF-α in the culture supernatants was measured using an enzyme-linked immunosorbent assay kit (Catalog # BMS607HS) for TNF-α (Invitrogen, Waltham, MA, USA)). The absorbance was measured using an ELISA reader at a wavelength of 450 nm. The relative TNF-α productions, with several concentrations of SPG, antisense TNF-α, and SPG-antisense TNF-α normalized to TNF-α production without them, were measured as the inhibition rate.

### 4.5. Real-Time PCR

RNA was extracted from the colon and CD11b+ cells using Trizol (Invitrogen, Carlsbad, CA, USA). The RNA was reverse transcribed into cDNA with ReverTra Ace qPCR RT Master Mix (Toyobo, Tokyo, Japan). The cDNA was quantified using real-time PCR (RT-PCR) with an ABI PRISM 7000 (Applied Biosystems Inc., Foster City, CA, USA). The RT-PCR for TNF-α, IL-1β, IL-6, dectin-1, and GAPDH was performed using the TaqMan probe. For the relative quantification of gene expression, the comparative Ct method was used. The final amount of the target gene, normalized to an endogenous reference gene as GAPDH (ΔCt = Ct target gene − Ct reference gene) was determined using the formula 2^−ΔCt^.

### 4.6. Flow Cytometric Analysis and Immunofluorescence

LPMCs were prepared in a phosphate-buffered saline buffer with 1% bovine serum albumin and 0.5% sodium azide, and were incubated with FITC labeled rat anti-mouse CD11b mAb (clone M1/70) and PE-labeled rat anti-mouse dectin-1 mAb (clone 2A11). The expression of CD11b and dectin-1 was examined via flow cytometry analysis (FACSVerse, BD Biosecience). CD11b+ cells isolated from LPMCs were incubated with Alexa546-labeled SPG-antisense TNF-α at different time points (0, 1, 2, and 4 h). The uptake of the complex was determined via flow cytometry. For immunofluorescence, CD11b+ cells were incubated with Alexa546-labeled antisense TNF-α or FITC labeled-SPG-Alexa546-antisense TNF-α for 4 h. Fluorescence microscope analyses were performed to detect antisense TNF-α or SPG-antisense TNF-α in CD11b+ cells using a BZ-9000 digital fluorescence microscope (Keyence, Osaka, Japan).

### 4.7. Treatment Using SPG-Antisense TNF-α

For therapeutic experiments in DSS-treated mice, 0.2 mg/kg of SPG, antisense TNF-α, or SPG-antisense TNF-α was administered to the colon via enema twice a week (days 0, 3, 7, and 10). To evaluate the activity of colitis, a video endoscopic system for mice was used (Karl Storz, Tuttlingen, Germany) [41]. The endoscopic findings in DSS-treated mice include the disappearance of a vascular pattern and the appearance of erosion and ulcers. A trained pathologist who was blind to the study evaluated the presence of colitis on day 14.

### 4.8. Statistical Analysis

The student *t*-test or the Mann–Whitney U test was used for the analysis of quantitative variables, and the chi-square test for qualitative variables. Two-sided *p* values of <0.05 were considered statistically significant.

## 5. Conclusions

TNF-α production both in vitro and in vivo was significantly inhibited by SPG-antisense TNF-α. The topical therapy by SPG-antisense TNF-α ameliorated intestinal inflammation. A more effective therapeutic effect can be expected using β-glucan-based drug delivery system that conjugates SPG and antisense. Our result demonstrated the possibility of new topical therapeutic approach against the inflammatory bowel disease.
